# Farmer Attitudes and Livestock Disease: Exploring Citizenship Behaviour and Peer Monitoring across Two BVD Control Schemes in the UK

**DOI:** 10.1371/journal.pone.0152295

**Published:** 2016-03-29

**Authors:** Claire Heffernan, Lena Azbel-Jackson, Joe Brownlie, George Gunn

**Affiliations:** 1 School of Veterinary Sciences, University of Bristol, Langford, Bristol, United Kingdom; 2 Livestock Development Group, University of Reading, Reading, United Kingdom; 3 Royal Veterinary College, North Mymms, Hatfield, Herts, United Kingdom; 4 Scotland’s Rural College, Aberdeen, United Kingdom; University of British Columbia, CANADA

## Abstract

The eradication of BVD in the UK is technically possible but appears to be socially untenable. The following study explored farmer attitudes to BVD control schemes in relation to advice networks and information sharing, shared aims and goals, motivation and benefits of membership, notions of BVD as a priority disease and attitudes toward regulation. Two concepts from the organisational management literature framed the study: citizenship behaviour where actions of individuals support the collective good (but are not explicitly recognised as such) and peer to peer monitoring (where individuals evaluate other’s behaviour). Farmers from two BVD control schemes in the UK participated in the study: Orkney Livestock Association BVD Eradication Scheme and Norfolk and Suffolk Cattle Breeders Association BVD Eradication Scheme. In total 162 farmers participated in the research (109 in-scheme and 53 out of scheme). The findings revealed that group helping and information sharing among scheme members was low with a positive BVD status subject to social censure. Peer monitoring in the form of gossip with regard to the animal health status of other farms was high. Interestingly, farmers across both schemes supported greater regulation with regard to animal health, largely due to the mistrust of fellow farmers following voluntary disease control measures. While group cohesiveness varied across the two schemes, without continued financial inducements, longer-term sustainability is questionable.

## Introduction

Bovine Viral Diarrhoea is a viral disease of cattle which impacts herd productivity and reproduction [1]. The disease is endemic in the UK with historical estimates of the overall cost i.e. control and prevention ranging from £25–60 million per annum [2]. In a study in Scotland, benefits to eradicating the disease at the national level were estimated to be £47 million in ‘discounted economic gain’ [3]. As BVD is an immunosuppressive disease and thereby may accelerate synergistic infections, calculating the economic impact is fraught with challenges. Relatively few studies have explored prevalence rates in the UK. In 2010, Brulisauer et al. estimated that 16% of beef suckler herds in Scotland had active BVDV infection [4].

Thus, at the macro-level, the eradication of BVD appears to be sensible and economically prudent. Nevertheless, the question remains given the apparent costs and benefits of eradication, why farmers have not embraced BVD control schemes? Over the past decade, a number of schemes have been implemented in the UK with various levels of success (where success is defined as improvements to the control of the disease). Previous studies among UK farmers have illustrated that livestock disease is largely perceived as an individual farmer problem and ‘good farmers’ do not suffer from animal health problems [5]. As such, collective behaviour is rare among UK farmers regarding disease control (ibid). Therefore, the authors evaluated two existing BVD control schemes (Orkney Livestock Association BVD Eradication Scheme and the Norfolk and Suffolk Cattle Breeders Association BVD Eradication Scheme). The Orkney scheme was initiated in 2001 with a grant secured from the local council for BVD testing so farmers would not incur costs for lab fees. By 2004, however, the testing subsidy was stopped. For the Norfolk/Suffolk scheme testing for the first 100 farmers enrolled in the scheme was free and for subsequent joiners, half price.

The two schemes varied dramatically in terms of group ‘cohesiveness’. Within the literature, cohesiveness is related to overall performance [6–14]. In this case, performance at the group level was related to the successful completion of a range of tasks related to BVD control at the community and/or regional level. Orkney farmers, as part of a close-knit Island community knew other members, while Norfolk scheme members did not. Further, the Orkney scheme was operationalised by the Orkney Livestock Association (OLA). Conversely, the Norfolk scheme was implemented by individual veterinary practices.

The authors examined elements of group behaviour to identify the factors potentially related to the success and failure of BVD control. Two constructs from the organizational management literature were applied: Group Organizational Citizenship Behavior (GOCB) [15–19] and ‘peer to peer’ monitoring [20–23]. Citizenship behaviour is defined as ‘individual behaviour that is discretionary, not directly or explicitly recognized by the formal reward system, and that in the aggregate promotes the effective functioning of the organization’ [24]. As such, GOCB is defined as a collective phenomenon that is based on a ‘normative or characteristic level of group member behaviour of supporting and helping each other’ [25]. In this context, ‘helping’ is defined as the cooperative and/or collaborative ‘processes’ along with the attendant attitudes, motivation and behaviours [26].

Thus, GOCB are considered the ‘extra-role’ behaviours that are voluntary and take place at the group level. Within the management literature ‘in role’ behaviours are those that may be measured by standard performance indicators in an organization while ‘extra role’ behaviours are ‘discretionary’ and not recognized by the organization itself [27]. Conversely, ‘peer to peer’ monitoring occurs when ‘individuals notice and respond to their peers’ behaviour or performance results’ [28]. As such, peer monitoring can constitute part of the ‘group level processes’, which make up GOCB. Within the literature, peer monitoring is considered a form of ‘informal control’, which can enhance organizational effectiveness (ibid).

Therefore, the aim of the study was to explore the role of GOCB and any associated peer monitoring across members vs. non-members of the two BVD schemes involved. Key ‘discretionary’ behaviours, which could enhance the effectiveness of the scheme were examined e.g. the transfer of information regarding BVD status and the sharing of animal health-related information between farmers. Critical to understanding such behaviour was first determining the strength and density of the social networks by which such information was transferred i.e. to whom and how often did farmers relay animal disease-related information. Other issues under evaluation included the level of compliance among scheme members and the motivational factors underpinning membership. While the studies of GOCB have examined the impact of particular leadership styles and other organisational factors such as the drivers to citizen behaviour, few studies have explored how perceptions regarding the authorities or institutions involved support or inhibit such behaviours.

Farmers are subject to a wide variety of regulations from the extra-national (EU) to the national to local levels. To determine the role that perceptions and attitudes toward such wider authorities may or may not play on GOCB, the authors drew upon the psychological aspects of Agency theory. According to the theory, an individual has two psychological states: the ‘autonomous state’ in which individuals make decisions on their own values, beliefs and experiences and the ‘agentic state’ in which decision-making is deferred to a higher authority [29]. With membership in a BVD scheme, individuals would go from an ‘autonomous’ state dealing with the disease to an ‘agentic’ state within the context of the scheme. And it is the effectiveness of the switch to the agentic state, which will be explored.

## Materials and Methods

In total, 162 farmers participated in the study. A range of non-probabilistic sampling strategies were utilized to target in-scheme members. Recommendations regarding initial scheme members to interview were made by the OLA manager. In this manner, the snow-ball technique was used to identify other known scheme members residing in an area of the initial interview [30]. To lower informant bias, ‘cold calling’ at the farm gate was utilised to interview those farmers who resided the study area, but who were not identified by the key informant in the first instance. Out of scheme farmers were also interviewed at the local cattle market (Orkney Auction Mart in Kirkwall). In the close-knit island community of Orkney, farmers reported high levels of social pressure to join the scheme. Therefore, farmers were often reluctant to classify themselves as a non-scheme member.

In Norfolk, local veterinarians involved in the scheme, identified scheme members. Latterly, a list of scheme farmers was supplied by the Royal Veterinary College (an implementer of the Norfolk/Suffolk scheme). Farmers were interviewed in person and via the telephone. Non-scheme farmers were interviewed at the Norwich Livestock Market, Norwich and Newark Livestock Market in Newark, Nottinghamshire. The markets were selected solely due to their geographic proximity to the schemes. Ultimately selection of the farmers across both schemes depended upon ‘convenience’ sampling or in this case, the good will of the individuals involved (Table 1). Data collection took place from January to March 2010. Procedures for data collection and participant consent were in full compliance with the ethical guidelines for research as detailed by the University of Reading Ethics Committee. Participants were shown the questionnaire from the first point of contact, when they were asked if they would like to participate in the study. Potential interviewees were also provided with a written statement detailing the nature of the research and confirmation that all responses would be anonymised. Those interested in participating were then asked to provide formal verbal consent to proceed with the questionnaire. Once consent was obtained, it was recorded on the individual’s questionnaire.

**Table 1:** Number of Participants by Scheme.

	In-scheme	Out of scheme
OLA	56	6
Norfolk/Suffolk	47	47

The data was analysed using the social networking software program, UCINET (Version 6.156, Analytic Technologies) [31] to explore the strength of social relationships and information transfer. Four features of the social networks were examined: ‘network density’ which explores the ‘interconnectedness’ of an actor network; the ‘degree measure’ which is the number of individual connections related to an actor; the ‘betweeness measure’ which is the extent an individual actor acted as a link or bridge to others and the ‘centrality’ or importance of different actors to the network. Key questions regarding authority figures and scheme membership were analysed utilising the software program Concordance (Version 3.3, R.J. Watt) to explore the discursive relationship surrounding key terms contained within the responses. Statistical analysis was performed using SPSS software (Version 22, IBM, 2014).

## Results

### Discretionary group behaviour: social networks and animal health information transfer

**Fig 1:**
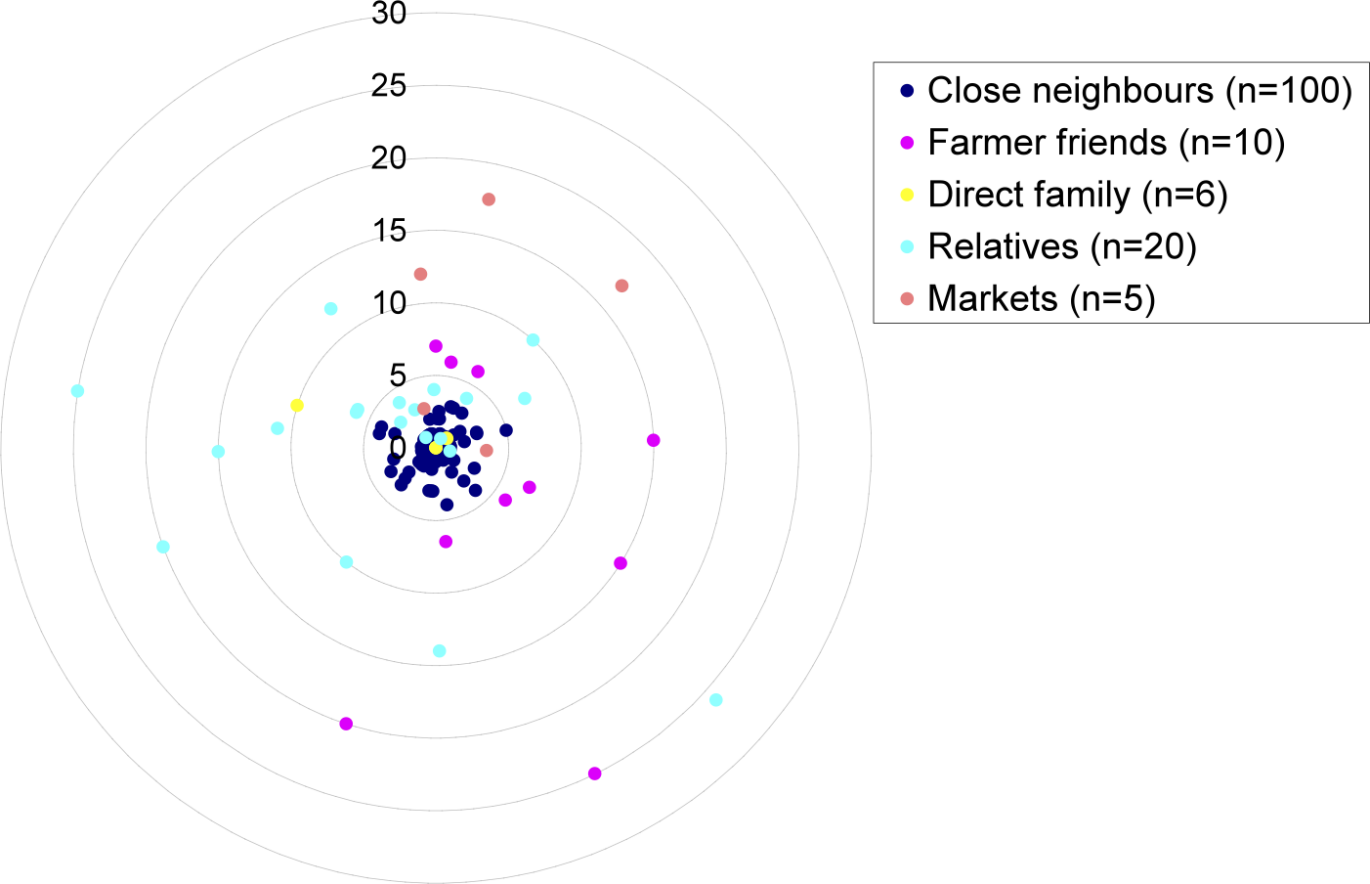
The Spatial Representation of Advice Networks (Orkney).

Animal health-related information was transferred via both formal and informal networks among both scheme members and non-members. A typology of the range of network relations was created based on geographical and relational data. For example, in Orkney, the social (informal) contacts with whom farmers shared advice, could be categorised into five main groups. By far the majority of networks were comprised of neighbours, who resided less then five miles away, often sharing property boundaries (as illustrated by the spatial representation of the social network of Orkney farmers, Fig 1). The remaining informal contacts were friends (living more than five miles away); direct family members (mainly children often living on the same farm); relatives (including indirect relations often living further away); and finally, other farmers (at the market place).

**Fig 2:**
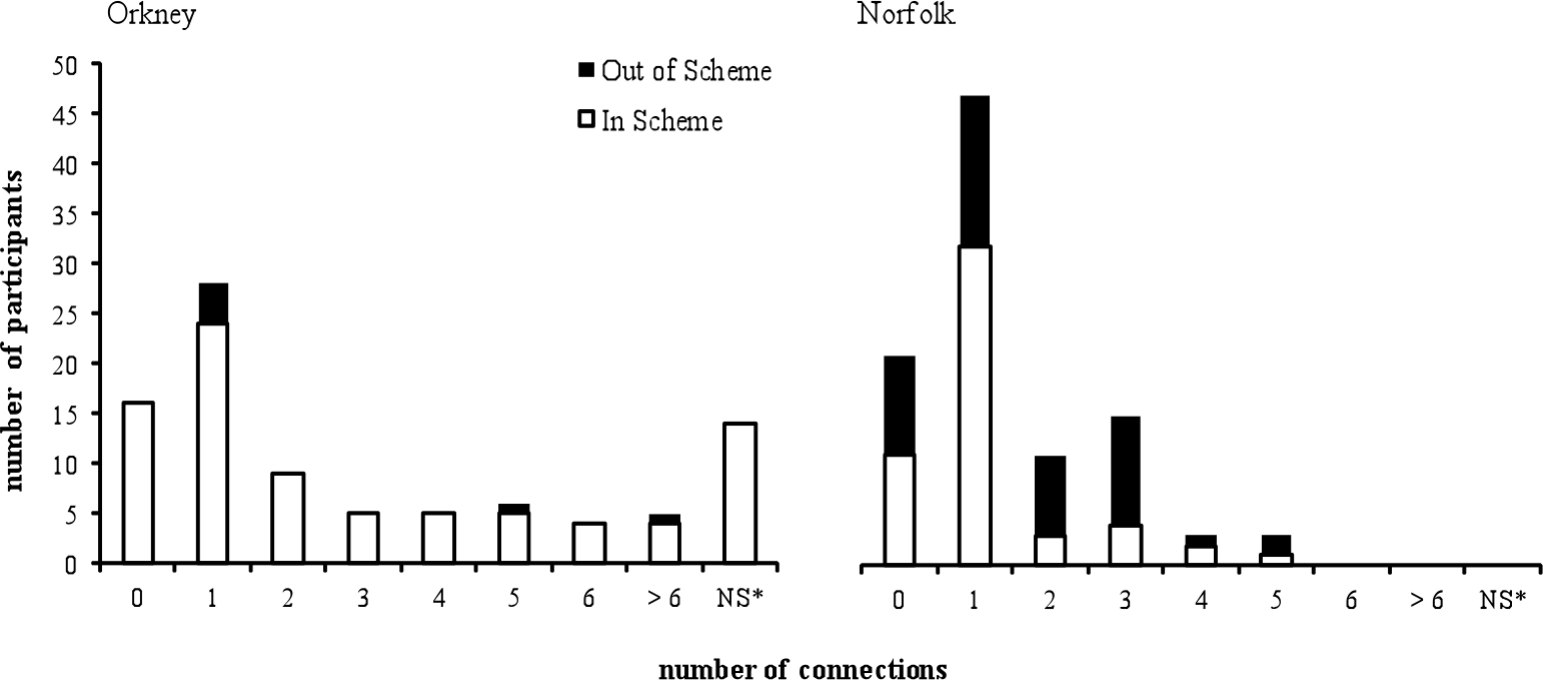
The Number (Proportion) of Social Connections Reported by Orkney and Norfolk Farmers.

The number of social connections varied by scheme (Fig 2).

To examine association between the scheme and the number of reported social connections a chi square statistical analysis was performed. The findings revealed no significant association between these variables *X*^*2*^(7) = 4.69, *p* > .05 (*p* = .70).

The proportion of farmers who reported having at least one social contact also did not differ between the in scheme (36%) and the out of scheme (37%) groups (Fig 2). Moreover, only a small proportion of farmers in the groups (9% in-scheme farmers and 17% of out of scheme farmers) reported having two social contacts. Further analysis revealed no difference in the number of social contacts between the in-scheme (*M* = 2.29, *SD* = 2.06) and the out of scheme (*M* = 2.18, *SD* = 1.42) farmers, *t* < 1.

Analysis of differences in the frequency of contacts with their social network members revealed that the Orkney farmers had more interactions with members of their social network (*M* = 2.31, *SD* = 2.45) than Norfolk farmers (*M* = 1.41, *SD* = 1.23), *p* < .05 (*p* = .014) (Fig 1). The findings suggest that both the frequency of contact and overall composition of social networks was influenced not by their participation in the scheme but by the type of community (i.e., close-knit vs. more socially isolated) in which they resided.

**Fig 3:**
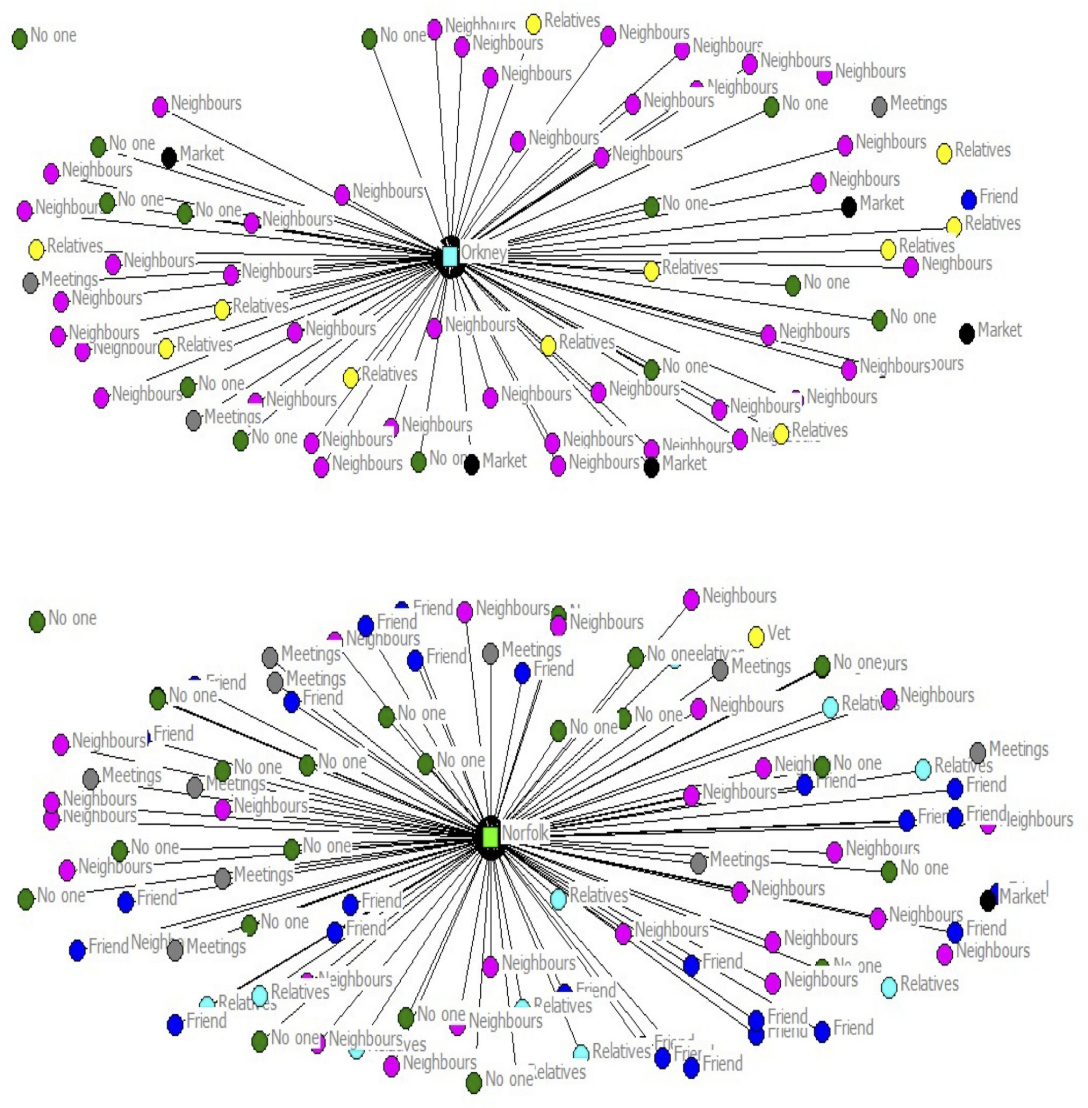
The Density of Information Sharing Networks (Orkney vs. Norfolk).

The density of the animal health information-sharing networks for the Orkney and Norfolk farmers also differed by geographic area (Fig 3). Neighbours were a more frequent source of advice in Orkney, while a greater number of Norfolk farmers exchanged information with friends at breed society meetings and other farmer gatherings.

The frequency in which these social contacts were consulted varied from two or three times a year to one or two times a week. Not surprisingly, relatives had the highest frequency of contact. Interestingly, other scheme members were not mentioned, as a specific source of advice. Scheme members were found to have different advice networks than non-scheme members (Table 2).

**Table 2:** Advice Networks by Scheme.

	% mentioned		
Social Group	Orkney scheme (N = 56)	Norfolk scheme (N = 47)	Out of scheme (N = 53)
**Market**	5	0	4
**Meetings**	3	8	11
**Neighbours**	44	36	25
**Relatives**	13	21	0
**Friends**	0	13	42
**Vets**	0	2	0
**No one**	16	20	19
**Not specified**	19	0	0
**Total**	100	100	100

Thus, overall scheme membership was related to sharing information on animal health with neighbours, while out of scheme farmers tended to report sharing information with friends.

The types of advice shared within farmer advice networks could be broadly categorised into the following: animal health, general farming and other information (Table 3).

**Table 3:** Types of information Shared Among the Different Social Groups.

% mentioned		
Social group	Animal health	General farming
Orkney scheme (N = 56)		
**Relatives**	2	1
**Neighbors**	2	6
**Farmer friends**	5	4
**Markets**	0	0
**Other**	2	2
**Not specified**	91	84
**Total**	100	100
Norfolk scheme (N = 47)		
**Relatives**	0	8
**Neighbors**	19	15
**Farmer friends**	6	2
**Markets**	6	0
**Not specified**	69	75
**Total**	100	100
Out of scheme (N = 53)		
**Relatives**	0	2
**Neighbors**	11	5
**Farmer friends**	21	23
**Markets**	4	9
**Not specified**	64	61
**Total**	100	100

**Fig 4:**
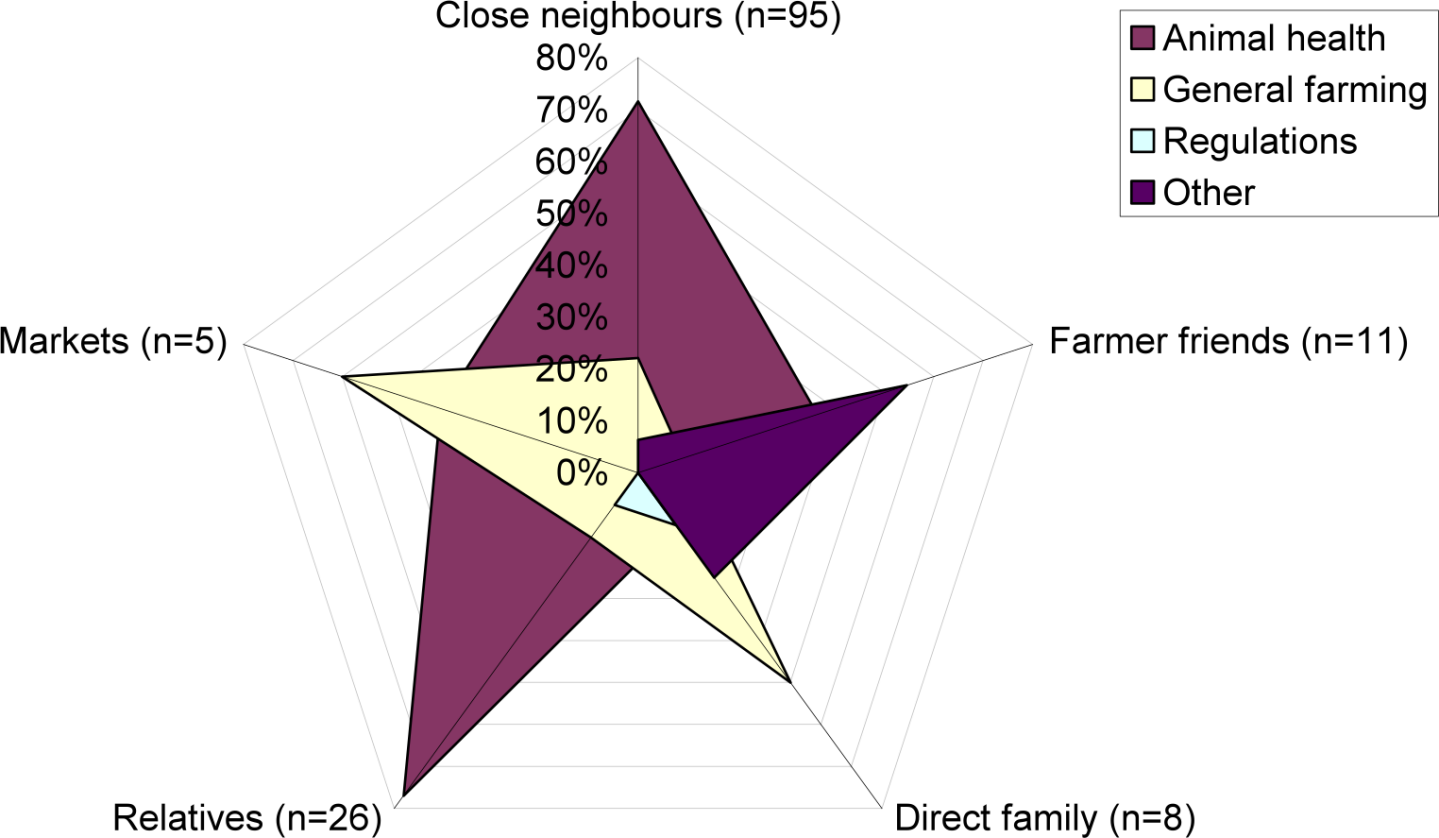
The Distribution of the Types of Advice Sought by Social Group.

As the table illustrates, animal health issues were mainly discussed/shared among close neighbours and relatives. The distribution of the type of advice sought among the different social groups by farmers in Orkney appeared as follows (Fig 4).

A number of farmers, however, noted that they never shared animal health information as it was considered ‘gossip’.

ID 73: “When speaking about animal health everything is general, while good neighbours will share this is isolated speaking about disease is a bit “iffy” people gossip.”

ID 71: “Animal health is like gossip it gets bigger and bigger. Talk about new regulations, not animal health gossip.”

As such, farmers noted that the disease information that was shared between contacts tended to be very general. However, a number of farmers on Orkney reported that discussing animal health issues had become more open with the advent of the BVD scheme.

ID70: “The BVD scheme has made everyone more open–it’s foolish to try to hide it.”

ID 72: “Talk about disease–[you] need good neighbours—some neighbours would tell you others won't—OLA helped making it open up.”

ID 69: “OLA is good for health and sending circulars. I learn a lot from talking to other farmers. OLA opened it up so that farmers can talk about disease.”

Nevertheless, only five farmers across the study set mentioned that they shared BVD status information with their friends and neighbours. Reasons for not disclosing BVD status largely revolved around the notion that BVD was a ‘dirty’ disease and hence a reflection of a farmer’s husbandry skills:

ID 96: “…farmers reluctant to share, if haven't got it they're not worried…They think will lose more than will gain—dirty farm syndrome. Pedigree breeders have valuable stock and don't want to disclose it.”

ID 80: “people don't like to take about BVD it’s a dirty disease.”

ID 88: “People worried who will use it [BVD status] in the future.”

ID 133: “[BVD is viewed]…in a bad light. We speak to each other, but wouldn't trust what they said though.”

Thus, despite being members of a BVD eradication scheme, the disease remained a sensitive subject.

### The groups: shared aims and goals

Membership in a group broadly entails that members share a common aim and will work together to meet that aim. Clearly in relation to a disease eradication scheme, it would be expected that group members would desire to be free of the disease on at least the farm level. However, across the study group, only a minority of farmers prioritised BVD as a problem (Table 4).

**Table 4:** Disease Priorities: Orkney vs. Norfolk Scheme Members vs. Out of Scheme Participants.

Desired Regulation	Orkney scheme (N = 56) (%)	Norfolk scheme (N = 47) (%)	Out of scheme (N = 53) (%)
**Abortion**	1	0	0
**Bluetongue**	1	6	4
**BVD**	16	4	12
**Fertility problems**	1	0	1
**Foot & Mouth Disease**	1	0	7
**Johnes disease**	6	0	7
**Leptospirosis**	3	0	3
**Mastitis**	0	2	1
**No priority**	47	68	10
**Pneumonia**	20	6	18
**Tuberculosis**	0	14	34
**Intestinal parasites**	1	0	1
**Total**	100	100	100

As the table illustrates, only 16% of scheme participants in Orkney rated BVD as a problem with only 4% of scheme members in Norfolk mentioning the disease. Reasons offered by farmers for the low ranking was the belief that the BVD is no longer a threat.

ID 45: “BVD is under control.”

ID 42: “BVD is almost sorted out now.”

ID 51: “All steps have now been taking to address the [BVD] problem.”

ID 22: “BVD is a problem of the past.”

Despite the lack of scientific evidence that the incidence and prevalence of BVD has declined dramatically, this was a meme among the farmers interviewed. Clearly, if the dominant perception is that the disease is no longer a concern, membership in the group over the longer-term is threatened. Therefore, in relationship to ‘shared aims’ to group formation on the most basic level, not all farmers viewed the impact of the disease in a similar manner. Consequently, not all farmers will share the same motivation for eradicating the disease. As the threat of the disease was not a key motivator for scheme membership other drivers must be involved.

### Motivation for membership

**Fig 5:**
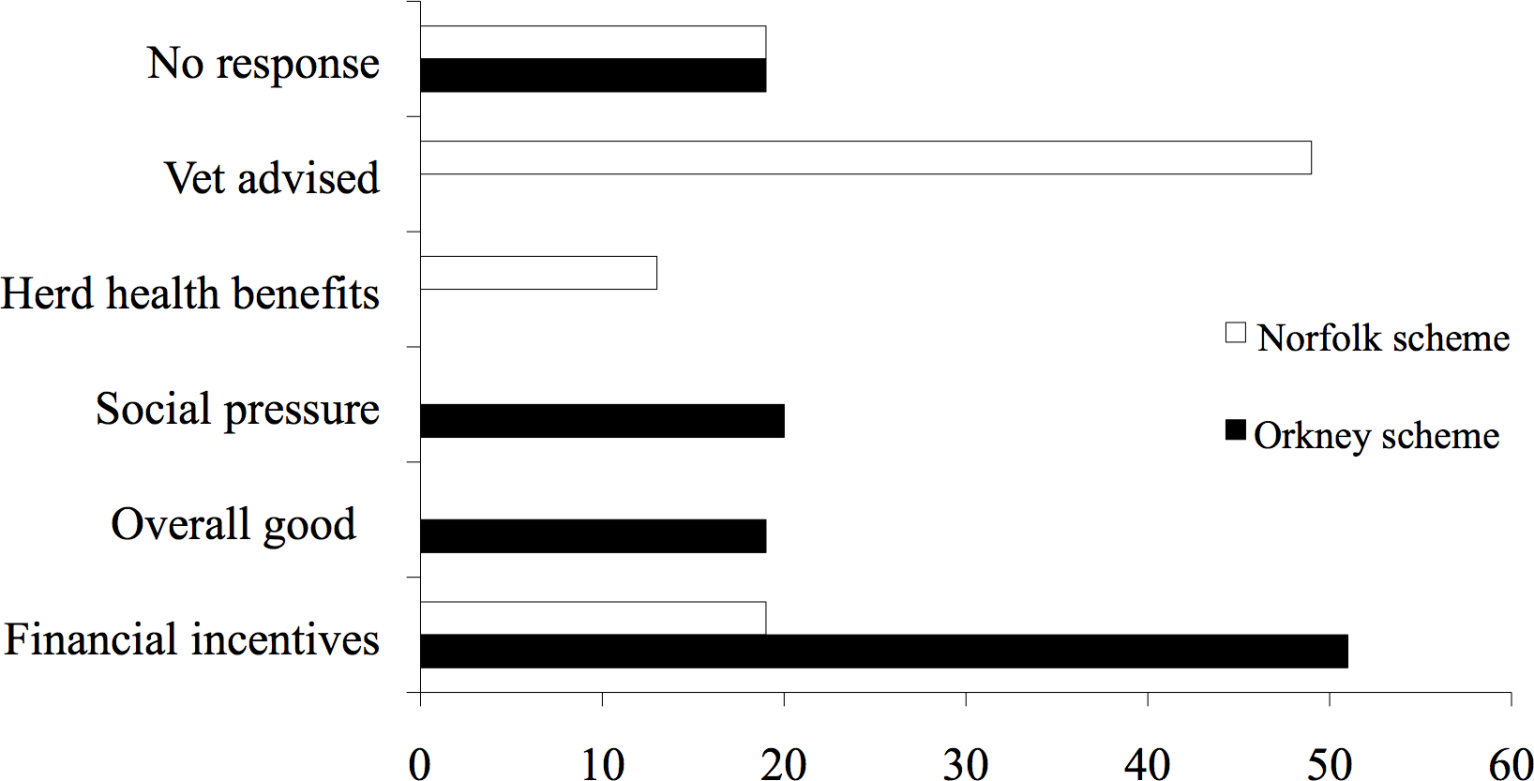
Motivation for Scheme Membership Reported by Orkney (solid black bars) vs. Norfolk Farmers (solid white bars).

In Orkney, motivation for scheme membership varied and a range of adopter groups could be discerned from the dataset. Participants offered a variety of reasons for joining the scheme such as: financial incentives (as noted above testing was free); external pressure (either by other farmers or by council members as an 80% joining rate had to be achieved to secure funding for the testing); non-adopters; for the overall good of the Island (to eradicate BVD from Orkney); and finally personal benefit (i.e. better herd health, lower costs, better sales etc). With regard to Norfolk, reasons for joining the scheme differed. Farmers tended to join either because their vet had advised them or to solve an existing or known prior problem with BVD. Financial reasons tended to focus on the subsidized testing. Nevertheless, many farmers in Norfolk found the question difficult to answer as many were of the opinion that they were not part of a group per se (Fig 5).

Statistical analysis established a significant association between the region and motivation for scheme membership *X*^*2*^(5) = 84.56, *p* < .05 (*p* = .0001). Over half of Orkney scheme members stated their reason for joining was for ‘personal gain’, as compared to more altruistic reasons such as the ‘overall good’ of the Island. The latter was a reason mainly given by those farmers who noted that they did not benefit directly from the scheme. Therefore, the incentive for these participants appeared to be social rather than economic. Nevertheless, a large number of farmers reported that they were no longer active in the scheme as once the subsidized testing programme finished, many farmers stopped testing. Indeed, at the time of the fieldwork while 5% of farmers noted they were ‘clear’, only 18% of farmers were testing on a regular basis. So although the farmers remained identified with the group, many were no longer active participants.

It is likely that reasons for joining the scheme would influence on a farmer’s decision to continue with the scheme over time. For example, one would expect farmers who joined due to external pressures, to cease their membership if such pressure was no longer there, unless other benefits were derived. Equally, for those who expected personal benefits, membership is likely to be discontinued if these expectations were not fulfilled.

### Perceived benefits of membership

Benefits of scheme membership differed across the study set (Table 5).

**Table 5:** Reported Benefits of Scheme Membership: Orkney vs. Norfolk.

Benefits of Scheme	Orkney scheme (N = 56)	Norfolk scheme (N = 47)
	**(%)**	**(%)**
**Indirect benefits**	3	7
**Better sale price**	7	2
**Easier to sell**	7	14
**Healthier animals**	28	16
**Improved herd performance**	1	5
**Increased fertility**	3	7
**Peace of mind**	2	14
**Reduced expense/Economic losses**	7	2
**Accreditation**	3	0
**No benefit**	22	32
**Reduced BVD in Orkney**	5	0
**Reduced risk when buying**	6	0
**No response**	6	0
**Total**	100	100

As the table illustrates, across both schemes, farmers noted a number of benefits of scheme membership ranging from economic gains to ‘peace of mind’. Nevertheless, a significant proportion also offered that membership had no benefits. Respondents who noted that the scheme had ‘no benefits’ crossed all of the above adopter groups in both geographic areas.

### Perceptions toward authority

In the vernacular of agency theory, study participants, in joining a disease scheme, move from an ‘autonomous’ state to an ‘agentic state’ in which membership rules and regulations would dominate. Nevertheless, such a move in the context of BVD schemes is voluntary and the agents involved are other farmers or community-level institutions.

On a wider national level, there is UK government support for the ‘beneficiary pays’ principle with regard to animal disease. The natural consequence of such an approach is that farmers shoulder a greater burden of the costs of animal health. In this manner, the government is supporting the reverse: a move from the ‘agentic’ state to the ‘autonomous’ state regarding livestock disease control among the communities involved.

Interestingly, however, study participants overwhelmingly desired compulsory regulation with regard to livestock disease (Table 6).

**Table 6:** Attitudes Toward Livestock Disease Regulation.

% Response			
Desired Regulation	Orkney scheme (N = 56)	Norfolk scheme (N = 47)	Out of scheme (N = 53)
**All compulsory**	56	53	52
**Mainly compulsory**	34	30	17
**Mainly voluntary**	8	15	13
**All voluntary**	2	2	19
**Total**	100	100	100

Across the groups, the desire for compulsory regulation regarding livestock diseases dominated. Further statistical analysis of the farmers responses showed no differences in the reported attitude ratings between the in scheme (*M* = 1.64, *SD* = 0.85) and the out of scheme (*M* = 1.93, *SD* = 1.2), *t*(160) = 1.763, *p* = .080 or between Orkney (*M* = 1.71, *SD* = 0.98) and Norfolk (*M* = 1.75, *SD* = 0.99), *t* < 1 (*t*(160) = -0.253, *p* = .801) participants.

When asked to explain their concerns, views tended to focus on mistrust of other farmers.

ID 19: If it is not enforced it would not work–always the odd one [farmer] that would not comply.

ID 37: If it is [disease regulation] voluntary there will be too many [farmers] who will dodge it [disease regulation]–everyone has to abide by it.

ID 146: Disease regulation has to be compulsory otherwise everyone will do as they please.

Thus, despite being part of a voluntary disease control group, scheme members still desired stronger regulation with regard to animal health. However, when asked to detail what other support for animal health is desirable, the majority of responses focused on greater financial support with less ‘red tape’. When asked to specify the particular diseases requiring such regulation, farmers were keen to devolve individual autonomy to the government for epidemic diseases but there was less consensus with regard to endemic diseases.

## Discussion

The results demonstrated that group ‘helping’ was not part of the construct of BVD scheme membership. Indeed, information transfer was limited regarding both sets of farmers and BVD status remained a sensitive subject. Although the schemes differed in the level of information sharing on the topic, farmer driven ‘citizenship behaviour’ was low. Peer to peer monitoring in the form of gossip regarding animal disease, however was high. Therefore, unhelpful peer monitoring was taking place, particularly in the relatively closed communities of Orkney. In relation to citizenship behaviour, there was little difference between in-scheme and out of scheme members or geographic area. Few farmers across the study set shared information as part of extra role behaviours.

From the outset, the two schemes varied widely with regard to ‘cohesiveness’. Interestingly, while ‘cohesiveness’ appeared to have a role in forging the group identity, it had little or no role in forging sustainability. Indeed, the results illustrated that while many Orkney farmers were no longer active participants in the group, they still identified strongly with the scheme. Conversely, many Norfolk farmers did not view themselves as part of a scheme in the first place.

Drivers for membership varied across the study set. However, while Orkney farmers initially bowed to social pressure from other farmers and scheme owners to join, the levels of active participants appeared to decline with the withdrawal of subsidized testing. Norfolk farmers were also responding to inducements of free or low cost tests from those in charge of running the scheme i.e. local vet practices. Across both groups a high percentage deemed the scheme had no benefits. The findings indicate that without further financial inducements, both schemes are likely to struggle over the longer term.

Clearly, joining a disease control scheme, on a variety of levels, devolves individual autonomy to the group. Each of the schemes involved had a variety of membership criteria i.e. attendance at training programs etc. with costs involved. Nevertheless, across the study group, while devolving individual authority in relation to epidemic disease was desirable, interference in endemic diseases was less clear-cut. Thus, future government policies based on the ‘beneficiary pays’ principle are likely to be strongly opposed by the farming community.

From the findings, a number of recommendations for the formation of farmer disease control groups can be made. First, the transient role and benefits of financial inducements in forging group membership should be recognised from the outset and alternate mechanisms be put in place when such inducements end. Second, perceptions regarding particular disease threats vary both in terms of social censure and overall economic impact. Again, both factors must be accounted for in the design and implementation of any future livestock disease control scheme. Finally, recognising the role that citizenship and agentic behaviour plays in preventing vs. aiding the control of particular disease threats is vital to improving the effectiveness and impact of both particular disease and wider herd health schemes.
